# Risk factors for mortality among patients undergoing major amputations due to infected diabetic feet

**DOI:** 10.1590/1677-5449.010717

**Published:** 2018

**Authors:** Natália Anício Cardoso, Ligia de Loiola Cisneros, Carla Jorge Machado, Ricardo Jayme Procópio, Túlio Pinho Navarro

**Affiliations:** 1 Universidade Federal de Minas Gerais – UFMG, Programa de Pós-graduação Ciências Aplicadas à Cirurgia e à Oftalmologia, Belo Horizonte, MG, Brasil.; 2 Universidade Federal de Minas Gerais – UFMG, Departamento de Fisioterapia, Belo Horizonte, MG, Brasil.; 3 Universidade Federal de Minas Gerais – UFMG, Departamento de Medicina Preventiva e Social, Belo Horizonte, MG, Brasil.; 4 Hospital Risoleta Tolentino Neves, Cirurgia Vascular, Belo Horizonte, MG, Brasil.; 5 Universidade Federal de Minas Gerais – UFMG, Departamento de Cirurgia, Belo Horizonte, MG, Brasil.

**Keywords:** diabetic foot, foot ulcer, infection, mortality

## Abstract

**Background:**

Foot ulcers in patients with diabetes are a major public health problem and are often associated with lower limbs amputation and mortality in this population.

**Objectives:**

To investigate the risk factors associated with mortality in patients with infected diabetic foot ulcers and major lower limb amputations.

**Methods:**

This was an observational, retrospective, case-control study with a sample of 78 patients with infected diabetic foot ulcers who had major lower limb amputations at a Vascular Surgery Service at a university hospital.

**Results:**

The mean age of the study sample was 63.8 ± 10.5 years, 54 (69.2%) were male, mean serum creatinine was 2.49 ± 2.4 mg/dL and mean serum hemoglobin was 7.36 ± 1.7 g/dL. There was a 47.4% rate of readmissions to the same hospital. Transtibial amputation was performed in 59.0%; and transfemoral amputation in 39.7% of the sample. In this sample, 87.2% had a positive culture, predominantly (68.0%) monomicrobial and nosocomial infection of ulcers was observed in 30.8%. The most common bacterial genera were *Acinetobacter* spp. (24.4%), *Morganella* spp. (24.4%) and *Proteus* spp. (23.1%). No bacterial genus was identified as a predictor of death. Creatinine level ≥ 1.3 mg/dL (OR 17.8; IC 2.1-150) and transfemoral amputation (OR 4.5; IC: 1.3-15.7) were associated with death.

**Conclusions:**

Serum creatinine levels ≥ 1.3 mg/dL and transfemoral amputation were risk factors for death.

## INTRODUCTION

 The diabetic foot is an important public health problem, 1 since it is the leading cause of hospital admission 2 and of hospital costs 1 of patients with diabetes mellitus. 

 The mortality rate among diabetic patients is high and 5 million deaths due to diabetes were registered in 2015. 3 Diabetic patients with foot ulcers have a mortality rate double that of diabetic patients without foot ulcers. 4

 Diabetic patients who undergo major amputation of a lower limb have low survival rates. 5 Around 10% of lower limb amputation patients die during the perioperative period. Within 1 year of amputation, 30% of patients die; in the third year the percentage rises to 50%; and mortality is 70% over 5 years. 6 These percentages may increase in developing countries, since patients tend to seek medical care when the ulcer infection is advanced, increasing the risk of amputation and death. 5

 Diabetic patients can have a range of serious comorbidities, such as advanced age, cardiac disease, coronary disease, cerebrovascular disease, renal failure, and respiratory failure. Patients with diabetic foot may also have neuropathy, deformities, ischemia, and infected ulcers. 7 Ulcer infections can be associated with mortality and high rates of non-traumatic lower limb amputations. 8

 According to the Infectious Diseases Society of America, 9 ulcer infections can be classified as mild, moderate, or severe. An infection is considered mild when there is no damage to tissue structures, such as muscle, tendon, bone, or joints. The moderate classification is attributed when there is involvement of tissue structures with risk of lower limb amputation. An infection is considered severe when, in addition to involvement of tissue structures, there is also generalized sepsis associated with severe hemodynamic and metabolic disorders and danger of patient death. 7 ^,^ 9

 Classifying the severity ulcer infections makes it possible to determine the correct treatment for the patient, which may be drug-based (antibiotic therapy) and/or surgical. Immediate and appropriate treatment of the infection can reduce its destructive effects and control the focus of sepsis. 10

 Infections considered acute and superficial are normally monomicrobial 11 and caused by aerobic Gram-positive cocci bacteria 11 ^-^ 14 ; and infections considered deep, chronic, or complicated predominantly involve Gram-negative bacteria. 8 ^,^ 14

 Infections of ulcers in diabetic feet are primarily polymicrobial, i.e. there is more than one bacterial species present. Gram-positive, Gram-negative, aerobic, and/or anaerobic bacteria may be isolated. 15 Polymicrobial infections can make healing of the ulcer less likely because of virulence factors secreted by the different species of bacteria present in the infection and, as a result, can lead to amputation and death. 12 Additionally, a subsequent reinfection of the ulcer can also cause delayed healing of surgical wounds, worsening the patient’s prognosis. 16

 This study was designed to investigate risk factors associated with mortality among patients with infected diabetic feet who underwent major amputations. 

## METHOD

 The study was approved by the Research Ethics Committee at the Universidade Federal de Minas Gerais (Ethics approval certificate: 33623414.6.00005149) and authorized by the Department for Teaching, Research, and Extension at the hospital in which the study was carried out. 

 This is an observational, retrospective, case-control study with a sample of 78 patients with diabetic feet and infected ulcers. All patients underwent major amputation (above the ankle) and were admitted via the vascular surgery service to the Hospital Risoleta Tolentino Neves, a tertiary University Hospital in Belo Horizonte (MG), Brazil, from January 2007 to December 2012. All patients had had bacteriological cultures of deep tissues. Patients whose biological specimens had been collected using a swab were not included in this study. 

 All open lesions on the feet of patients with diabetes were defined as ulcers. All ulcers analyzed were deep, graded as ≥ 3 on the Wagner Classification, which corresponds to deep ulcers with formation of abscess or bone involvement, with localized gangrene or extensive gangrene. 

 No specific tests for neuropathy were conducted, but all of the patients in this sample had signs and symptoms compatible with diabetic neuropathy and lesions typical of the diabetic foot (in areas with sensory dysfunction and excessive mechanical stress). The medical team conducted vascular assessments by means of palpation of distal pulses, measurement of the ankle-brachial index, using Doppler ultrasound, but these data are not evaluated in this study. 

 Cultures in which there was no bacterial growth were considered negative. Cultures that grew two or more different bacteria were considered polymicrobial. A nosocomial infection was considered to have occurred if different bacterial genera were isolated in deep tissue cultures collected from the same ulcer at different times during the same hospital stay. 17

 The following variables were taken from the patients’ electronic medical records: age, sex, surgeries performed, deaths, serum hemoglobin and creatinine test results and results of bacterial cultures (including bacterial genera). 

 The case group comprised patients who had a major amputation and died; and the control group comprised the surviving patients who underwent a major amputation. 

 Statistical analyses were performed using Stata/SE 12.0 for Mac. Continuous variables were expressed as mean and standard deviation and analyzed using Student’s *t* test; categorical variables were analyzed using Pearson’s chi-square test or Fisher’s test (if the expected number of cases in one of the categories was less than 5). Risk factors for death after major amputation were identified using logistic regression analyses. Values that were statistically significant according to the Wald test (univariate analysis) were included in a multivariate model, and the final model was obtained by sequential removal of variables based on the results of the Wald test and the Hosmer and Lemeshow test. All models are shown. Statistical significance for selection of the final model was estimated using the Hosmer and Lemeshow test (p < 0.05). 

## RESULTS

Table 1 lists the data for the study variables from the 78 patients in the case-control study, both as the entire sample and separated by group and also comparisons between them. 

**Table 1:** Comparison between study variables for patient sample and subsets.

	**Total** **(n = 78)**	**Major amputation and death** **(n = 18)**	**Major amputation and no death** **(n = 60)**	**p**
Age, Mean (SD)	63.8 (10.5)	68.3 (11.4)	62.4 (9.9)	0.034 ^*^
Male n (%)	54 (69.2)	11 (61.1)	43 (71.7)	0.395
Elevated serum creatinine, Mean (SD)	2.49 (2.4)	3.65 (2.6)	2.14 (2.3)	0.019*
Low serum hemoglobin, Mean (SD)	7.36 (1.7)	6.48 (1.7)	7.61 (1.6)	0.011*
Number of cultures per patient n (%) 1 culture 2 cultures > 2 cultures	42 (53.9) 21 (26.9) 15 (19.2)	10 (55.6) 5 (27.8) 3 (16.7)	32 (53.3) 16 (26.7) 12 (20.0)	0.999
Number of patients with positive cultures n (%)	68 (87.2)	17 (94.4)	51 (85.0)	0.438
Number of patients with polymicrobial culture n (%)	25 (32.1)	6 (33.3)	19 (31.7)	0.894
Number of patients with nosocomial ulcer infection n (%)	24 (30.8)	4 (22.2)	20 (33.3)	0.561
Number of patients readmitted n (%)	37 (47.4)	8 (44.4)	29 (48.3)	0.772
Number of major amputations n (%) Transtibial Transfemoral Hip disarticulation	46 (59.0) 31 (39.7) 1 (1.3)	6 (33.3) 11 (61.1) 1 (5.6)	40 (66.7) 20 (33.3) 0 (0.0)	0.010*

* p < 0.05; SD = standard deviation.

 Mean of age of the entire sample was 63.8 years (standard deviation [SD] 10.5), and 54 (69.2%) of them were male. The major amputation patients who died had higher mean serum creatinine levels – 3.65 mg/dL (SD 2.6) – and lower mean serum hemoglobin levels – 6.48 g/dL (SD 1.7) – when compared with major amputation patients who did not die. Approximately 53% of the patients were only admitted once, while 37 patients (47.4%) needed to be admitted more than once. Transtibial amputations were performed on 59.0% of the patients and transfemoral amputations on 39.7%. Although the majority of patients (59.0%) had transtibial amputations, transfemoral amputation (61.1%) was associated with death (p = 0.010). 

 Sixty-eight of the 78 patients (87.2%) had positive cultures and 12.8% had negative cultures. During the reference admission, 42 patients (53.9%) had just one bacterial culture from deep tissue samples, 21 (26.9%) had two cultures, and 15 (19.2%) had more than two cultures. Of the 68 patients with positive cultures, 67.9% of cultures grew just one microorganism, while 32.1% grew two or more different microorganisms. Nosocomial ulcer infection was recorded for 24 patients (30.8%). 

Table 2 lists the bacterial genera isolated from bacterial cultures of deep tissue specimens from this sample of patients. The bacteria most frequently isolated from the subset of major amputation patients who died were *Acinetobacter* spp. (33.3%), *Morganella* spp. (33.3%), and *Proteus* spp. (27.8%). 

**Table 2:** Comparison between groups of patients by genera of bacteria isolated in bacterial cultures from deep tissues.

	**Total** **(n = 78)**	**Major amputation and death** **(n = 18)**	**Major amputation and no death** **(n = 60)**	**p**
Gram-positive				
*Enterococcus* spp. n (%)	15 (19.2)	3 (16.7)	12 (20.0)	0.999
*Staphylococcus* spp. n (%)	8 (10.3)	2 (11.1)	6 (10.0)	0.999
*Streptococcus* spp. n (%)	4 (5.1)	1 (5.6)	3 (5.0)	0.999
Gram-negative Genera				
*Acinetobacter* spp. n (%)	19 (24.4)	6 (33.3)	13 (21.7)	0.312
*Citrobacter* spp. n (%)	4 (5.1)	1 (5.6)	3 (5.0)	0.999
*Escherichia* spp. n (%)	9 (11.5)	2 (11.1)	7 (11.7)	0.999
*Enterobacter* spp. n (%)	9 (11.5)	2 (11.1)	7 (11.7)	0.999
*Klebsiella* spp. n (%)	6 (7.7)	0 (0.0)	6 (10.0)	0.327
*Morganella* spp. n (%)	19 (24.4)	6 (33.3)	13 (21.7)	0.312
*Proteus* spp. n (%)	18 (23.1)	5 (27.8)	13 (21.7)	0.589
*Pseudomonas* spp. n (%)	13 (16.7)	2 (11.1)	11 (18.3)	0.721
*Serratia* spp. n (%)	4 (5.1)	1 (5.6)	3 (5.0)	0.999
*Stenotrophomonas* spp. n (%)	1 (1.3)	0 (0.0)	1 (1.7)	0.999

Frequencies were calculated using the entire sample of 78 patients.

Table 3 lists the results of the univariate analysis, with indicated that the following variables were significant: age, creatinine levels ≥ 1.3 mg/dL, hemoglobin levels < 11 g/dL, and transfemoral amputation. 

**Table 3:** Univariate analysis of 23 clinical, laboratory, and bacterial variables.

	**Odds Ratio**	**95% confidence interval**	**p**
Age, per year	1.06	1.00-1.12	0.040 ^*^
Genus/gender, male	1.60	0.53-4.84	0.397
Creatinine ≥ 1.3 mg/Dl	15.9	1.99-127.2	0.009*
Hemoglobin < 11 g/dL	0.63	0.43-0.92	0.015*
Positive cultures	3.00	0.35-25.44	0.314
Polymicrobial culture	1.08	0.35-3.31	0.894
Rate of nosocomial ulcer infections	0.57	0.17-1.96	0.374
Rehospitalization	0.86	0.29-2.46	0.772
Transfemoral amputation	3.67	1.18-11.35	0.024*
*Acinetobacter* spp.	1.80	0.57-5.75	0.316
*Citrobacter* spp.	1.12	0.11-11.45	0.925
*Escherichia* spp.	0.95	0.18-5.02	0.948
*Enterococcus* spp.	0.80	0.20-3.22	0.753
*Enterobacter* spp.	0.95	0.18-5.02	0.948
*Klebsiella* spp.^£^	---	---	---
*Morganella* spp.	1.81	0.57-5.75	0.316
*Proteus* spp.	1.39	0.42-4.62	0.590
*Pseudomonas* spp.	0.56	0.11-2.78	0.476
*Staphylococcus* spp.	1.13	0.21-6.13	0.892
*Serratia* spp.	1.12	0.11-11.45	0.925
*Stenotrophomonas* spp.	---	---	---
*Streptococcus* spp.	1.12	0.11-11.45	0.925

* p < 0.05;

*^£^*for *Klebisiella* spp. and *Stenotrophomonas* spp*.,* there were no deaths.

Table 4 lists the results of the multivariate analysis, showing which factors were associated with death in this sample. 

**Table 4:** Multivariate analysis of variables associated with patient deaths.

	**Odds Ratio**	**95% confidence interval**	**p**
Creatinine ≥ 1.3 mg/dL	17.8	2.1-149.5	0.008
Transfemoral amputation	4.5	1.3-15.7	0.016

## DISCUSSION

 This study investigated risk factors associated with mortality in patients with infected diabetic feet who underwent major amputations. Serum creatinine greater than or equal to 1.3 mg/dL was considered a risk factor for death, as was transfemoral amputation. 

 In this study, patients with serum creatinine greater than or equal to 1.3 mg/dL had a risk of death almost 17.8 times that of patients with creatinine levels below this cutoff. Diabetic patients with nephropathy develop feet ulcers at double the rate of other diabetics. Among patients with diabetic nephropathy, the presence of ulcers on the feet is considered one of the primary causes of morbidity. These patients’ risk of amputation is 6.5 to 10 times greater, because the time taken for ulcers to heal is prolonged when serum creatinine is elevated. Additionally, mortality is associated with the severity of diabetic nephropathy. 18 ^-^ 20

 In this sample, 39.7% of patients had a transfemoral amputation and 61.1% of this subset died. These patients exhibited a risk of death 4.5 times that of patients who had amputations at other levels. A diabetic patient has a 15 to 40 times greater risk of amputation than a person who is not diabetic. Transfemoral amputation patients manifest serious systemic diseases, such as heart failure, coronary artery disease, cerebrovascular disease, renal failure, and chronic obstructive pulmonary disease, among others. They therefore have a high mortality rate. 21

 The bacterial genus isolated from infected ulcers of diabetic feet was not a risk factor associated with mortality. The bacterial genera *Acinetobacter* spp. (33.3%), *Morganella* spp. (33.3%), and *Proteus* spp. (27.8%), all part of the Gram-negative group of bacteria, were the types most frequently isolated in cultures from the major amputation patients who died. 

 A study conducted at the Hospital Central, Santa Casa de São Paulo, Brazil, also observed isolation of Gram-negative bacteria, with 66% of cultures from deep ulcers (Wagner Classification ≥ 3) growing Gram-negative bacteria. 22

 In the sample studied here, 12.8% of patients had a negative deep tissue culture, i.e., no bacteria were isolated. It should be noted that many of the patients studied would have already been treated with antibacterial drugs at other health centers, and use of antibacterials before collection of the biological material can cause a negative culture, masking the true result. 8

 Polymicrobial cultures may be present in infections of deep ulcers in patients with diabetic foot. 11 A patient with polymicrobial infection exhibits a slower ulcer healing process due to the presence of many virulence factors secreted by the different species of bacteria present in the infection. 12 In this study, predominance of polymicrobial cultures (32.1%) was not observed and this variable was not considered a risk factor for death. 

Certain factors can contribute to monomicrobial cultures.

 Patients with recent ulcers, i.e., those that have been developing for a short period of time, tend to manifest monomicrobial bacteria isolation. 23 Since this is a retrospective study, it was not possible to determine the time since ulcer onset in these patients, because this detail was not recorded in the electronic patient records. 

 Use of antibacterials by patients before samples are taken can also lead to isolation of only those bacteria that are resistant to the antibacterial, since bacteria that were susceptible to the prior treatment will have been eliminated. 23 Many of the patients studied had been admitted to the institution already taking antibacterials prescribed at other healthcare centers. 

 The result of a culture of biological material can also be influenced by incorrect sample collection from the ulcer. The best specimens for analysis by culture would be collected from deep tissues free from contamination or necrosis, since collections from superficial tissues can reduce the number of pathogens isolated, resulting in monomicrobial cultures. 24

 Finally, limitations at the microbiology laboratory used should also be considered, since it cannot perform cultures of obligate anaerobes. Cultures for facultative anaerobes are primarily ordered in osteomyelitis cases. For this patient sample, there were no records of cultures of facultative anaerobes. Anaerobic bacteria are present in 15% of severe infections of diabetic foot ulcers. 11

 In this study, the rate of nosocomial ulcer infections (30.8%) was not a risk factor for death. These infections may be related to contamination by hospital equipment or by the medical team’s hands, which could be colonized and come into direct contact with patients. 

 In this sample, male patients were the majority (69.2%) and mean age was over 60 years. Patients who died (68.3 years, SD 11.4) had a higher mean age than those who survived (62.4 years, SD 9.9) (p = 0.034). Advanced age has been identified as a risk factor for increased hospital mortality among patients who have major amputations preceded by lower limb ulcers. 5

 These patients had a 47.4% rehospitalization rate, but this elevated rate was not associated with death. In the majority of cases, the patient returned to the institution due to infectious complications related to the amputation stump. 25

 This study is subject to certain limitations. First, the lack of data on certain variables is inevitable and is considered a characteristic of retrospective studies. The time since ulcer onset, considered an important factor, was not recorded. Another relevant factor is the laboratory’s limitations with relation to performing cultures of anaerobes. Furthermore, many tissue samples were taken from patients who may have been taking antibacterials prior to admission. 

 In conclusion, serum creatinine levels ≥ 1.3 mg/dL and transfemoral amputation were associated with death in patients amputated due to infected diabetic feet. 
