# Supplementary material for: Risk factors for mortality among patients undergoing major amputations due to infected diabetic feet
Source: J Vasc Bras. 2018 Oct-Dec;17(4):296–302. doi: 10.1590/1677-5449.010717 (PMC6375264; doi:10.1590/1677-5449.010717)
Supplement: Translation

# Translation: Fatores de risco para mortalidade em pacientes submetidos a amputações maiores por pé diabético infectado

**Authors:** Natália Anício Cardoso, Ligia de Loiola Cisneros, Carla Jorge Machado, Ricardo Jayme Procópio, Túlio Pinho Navarro

**Affiliations:** 1 Universidade Federal de Minas Gerais – UFMG, Programa de Pós-graduação Ciências Aplicadas à Cirurgia e à Oftalmologia, Belo Horizonte, MG, Brasil.; 2 Universidade Federal de Minas Gerais – UFMG, Departamento de Fisioterapia, Belo Horizonte, MG, Brasil.; 3 Universidade Federal de Minas Gerais – UFMG, Departamento de Medicina Preventiva e Social, Belo Horizonte, MG, Brasil.; 4 Hospital Risoleta Tolentino Neves, Cirurgia Vascular, Belo Horizonte, MG, Brasil.; 5 Universidade Federal de Minas Gerais – UFMG, Departamento de Cirurgia, Belo Horizonte, MG, Brasil.

**Keywords:** pé diabético, úlcera do pé, infecção, mortalidade

## Abstract

**Contexto:**

A lesão no pé de pacientes com diabetes é um importante problema de saúde pública que frequentemente está associado a amputações em membros inferiores e mortalidade nessa população.

**Objetivos:**

Investigar os fatores de risco associados a mortalidade em pacientes com pé diabético infectado submetidos a amputação maior.

**Métodos:**

Estudo observacional, retrospectivo e caso-controle. Amostra composta por 78 pacientes com pé diabético e úlcera infectada submetidos a amputação maior em um serviço de cirurgia vascular em um hospital universitário no período de 5 anos.

**Resultados:**

A média de idade da amostra estudada foi de 63,8 ± 10,5 anos, com 54 (69,2%) pacientes do sexo masculino, com creatinina sérica média de 2,49 ± 2,4 mg/dL e hemoglobina sérica média de 7,36 ± 1,7 g/dL. Houve 47,4% de reinternação. Foi realizada amputação transtibial em 59,0% e transfemoral em 39,7% da amostra estudada. Nesta amostra, 87,2% dos pacientes apresentaram cultura positiva, predominantemente monomicrobiana (67,9%), e 30,8% apresentaram infecção hospitalar da úlcera. Os gêneros de bactérias mais frequentes foram *Acinetobacter* spp. (24,4%), *Morganella* spp. (24,4%) e *Proteus* spp. (23,1%). Nenhum gênero bacteriano foi identificado como fator de risco para óbito. O nível de creatinina ≥ 1,3 mg/dL (OR 17,8; IC 2,1-150) e a amputação transfemoral (OR 4,5; IC: 1,3-15,7) foram fatores de risco para o óbito.

**Conclusões:**

Os níveis séricos de creatinina ≥ 1,3 mg/dL e amputação transfemoral foram fatores de risco para óbito.

## INTRODUÇÃO

 O pé diabético é um importante problema de saúde pública 1 , pois é a principal causa de internação 2 e de gastos hospitalares 1 de pacientes com diabetes mellitus. 

 A taxa de mortalidade em pacientes diabéticos é alta, com 5 milhões de mortes decorrentes ao diabetes registrado em 2015 3 . Pacientes diabéticos com úlceras nos pés apresentam uma taxa de mortalidade duas vezes maior quando comparada com a de pacientes diabéticos sem úlceras nos pés 4 . 

 Pacientes diabéticos que foram submetidos a amputação maior do membro inferior apresentam baixas taxas de sobrevida 5 . Cerca de 10% dos pacientes que amputam o membro inferior morrem no período perioperatório. No primeiro ano após a realização da amputação, 30% dos pacientes evoluem a óbito; no terceiro ano, esse percentual sobe para 50%; e no quinto ano, são 70% 6 . Essa porcentagem pode aumentar em países em desenvolvimento, pois os pacientes tendem a procurar assistência médica quando a infecção da úlcera está avançada, o que aumenta o risco de amputação e óbito 5 . 

 O paciente diabético pode apresentar várias comorbidades graves, como idade avançada, doença cardíaca, doença coronariana, doença cerebrovascular, insuficiência renal e insuficiência respiratória. Além dessas, o paciente com pé diabético pode apresentar neuropatia, deformidades, isquemia e infecção da úlcera 7 . A infecção da úlcera pode se associar a mortalidade e a altas taxas de amputação não traumática dos membros inferiores 8 . 

 Segundo a *Infectious Diseases Society of America* 9 , a infecção da úlcera pode ser classificada como leve, moderada e grave. A infecção é considerada leve quando não há comprometimento de estruturas teciduais, como músculo, tendão, osso ou articulação. A classificação moderada é atribuída quando há comprometimento dessas estruturas teciduais com risco de amputação do membro inferior. A infecção é considerada grave quando, além do comprometimento das estruturas teciduais, existe a associação de sepse generalizada às alterações hemodinâmicas e metabólicas graves, com risco de levar o paciente a óbito 7 ^,^ 9 . 

 Classificar a gravidade da infecção da úlcera permite determinar o tratamento adequado para o paciente, que pode ser medicamentoso (antibioticoterapia) e/ou cirúrgico. O tratamento imediato e adequado da infecção pode diminuir seu efeito destrutivo e controlar o foco séptico 10 . 

 Infecções consideradas superficiais e agudas são normalmente monomicrobianas 11 e causadas por bactérias aeróbias cocos Gram-positivas 11 ^-^ 14 ; e infecções consideradas profundas, crônicas ou complicadas apresentam predomínio de bactérias Gram-negativas 8 ^,^ 14 . 

 As infecções das úlceras em pés diabéticos são principalmente polimicrobianas, ou seja, há presença de mais de uma espécie bacteriana. Bactérias Gram-positivas, Gram-negativas, aeróbias e/ou anaeróbias podem ser isoladas 15 . Infecções polimicrobianas podem dificultar a cicatrização da úlcera devido a fatores de virulência que são secretados pelas diferentes espécies de bactérias presentes na infecção e, por conseguinte podem levar a amputação e óbito 12 . Além disso, uma nova infecção da úlcera também pode causar atraso na cicatrização cirúrgica e piora no prognóstico do paciente 16 . 

 Este estudo visou investigar os fatores de risco associados a mortalidade em pacientes com pé diabético infectado submetidos a amputação maior. 

## MÉTODO

 Este estudo foi aprovado pelo Comitê de Ética em Pesquisa da Universidade Federal de Minas Gerais (CAAE: 33623414.6.0000.5149) e autorizado pelo Núcleo de Ensino, Pesquisa e Extensão do hospital em que o estudo foi realizado. 

 Trata-se de um estudo observacional, retrospectivo e caso-controle realizado em uma amostra composta por 78 pacientes com pé diabético e lesão ulcerada com infecção. Os pacientes foram submetidos a amputação maior (porção acima do tornozelo) no Serviço de Cirurgia Vascular do Hospital Risoleta Tolentino Neves, um hospital universitário terceirizado, em Belo Horizonte (MG), Brasil, no período de janeiro de 2007 a dezembro de 2012. Todos os pacientes possuíam cultura bacteriológica de tecido profundo. Não foram incluídos no estudo os pacientes que realizaram coleta do material biológico com *swab*. 

 Toda lesão aberta no pé dos pacientes com diabetes foi considerada úlcera. As lesões ulceradas analisadas foram todas profundas, graduadas como ≥ 3 segundo a Classificação de Wagner, o que corresponde a úlcera profunda com formação de abscesso ou envolvimento ósseo, com gangrena localizada ou com gangrena extensa. 

 Não foram realizados testes específicos para neuropatia, mas todos os pacientes dessa amostra apresentavam sintomas e sinais próprios da neuropatia diabética e das lesões típicas do pé diabético (em área com disfunção sensorial e hiperpressão mecânica). A avaliação vascular realizada pela equipe médica foi feita por palpação de pulsos distais e medida do índice tornozelo-braquial realizadas com o Doppler ultrassônico, contudo, essa medida não foi avaliada neste estudo. 

 Nas culturas em que não houve crescimento de bactérias, a amostra foi considerada negativa. As culturas em que houve crescimento de duas ou mais bactérias foram consideradas polimicrobianas. Foi considerado como infecção hospitalar da úlcera o isolamento de diferentes gêneros bacterianos em culturas de tecidos profundos realizadas em momentos distintos de uma mesma internação 17 . 

 Foram coletadas as seguintes variáveis após o acesso ao prontuário eletrônico dos pacientes: idade, sexo, cirurgias realizadas, evolução para o óbito, resultados de hemoglobina e creatinina séricas e resultados das culturas bacteriológicas (gêneros bacterianos). 

 O grupo-caso foi composto por pacientes submetidos a amputação maior que morreram; e o grupo-controle foi composto por pacientes submetidos a amputação maior que não morreram. 

 Para análise estatística, foi usado o programa Stata/SE 12.0 for Mac. Variáveis contínuas foram expressas em média e desvio padrão e foram analisadas usando test t de Student; as variáveis categóricas foram analisadas usando o qui-quadrado de Pearson ou teste de Fisher (se o número esperado de casos em uma categoria fosse inferior a 5). Os fatores de risco para óbito após amputação maior foram determinados por meio de análises de regressão logística. Os valores que foram estatisticamente significativos no teste de Wald (análise univariada) foram incluídos em um modelo multivariado, e o modelo final foi obtido por deleção sequencial de variáveis com base nos testes de Wald e Hosmer e Lemeshow. Todos os modelos foram apresentados. A significância estatística para a seleção do modelo final foi aferida pelo teste de Hosmer e Lemeshow (p < 0,05). 

## RESULTADOS

 Na Tabela 1 , estão apresentados os dados das variáveis estudadas dos 78 pacientes do estudo caso-controle referentes à amostra total e separados por grupo, além da comparação entre eles. 

**Tabela 1:** Comparação entre os grupos de pacientes das diferentes variáveis estudadas.

	**Total** **(n = 78)**	**Amputação maior e óbito** **(n = 18)**	**Amputação maior e não óbito** **(n = 60)**	**p**
Idade, média (DP)	63,8 (10,5)	68,3 (11,4)	62,4 (9,9)	0,034 ^*^
Sexo masculino n (%)	54 (69,2)	11 (61,1)	43 (71,7)	0,395
Maior nível de creatinina sérica, média (DP)	2,49 (2,4)	3,65 (2,6)	2,14 (2,3)	0,019*
Menor nível de hemoglobina sérica, média (DP)	7,36 (1,7)	6,48 (1,7)	7,61 (1,6)	0,011*
Número de culturas realizadas por paciente n (%) 1 cultura 2 culturas > 2 culturas	42 (53,9) 21 (26,9) 15 (19,2)	10 (55,6) 5 (27,8) 3 (16,7)	32 (53,3) 16 (26,7) 12 (20,0)	0,999
Número de pacientes com culturas positivas n (%)	68 (87,2)	17 (94,4)	51 (85,0)	0,438
Número de pacientes com cultura polimicrobiana n (%)	25 (32,1)	6 (33,3)	19 (31,7)	0,894
Número de pacientes com infecção hospitalar da úlcera n (%)	24 (30,8)	4 (22,2)	20 (33,3)	0,561
Número de pacientes com reinternação n (%)	37 (47,4)	8 (44,4)	29 (48,3)	0,772
Número de amputação maior n (%) Transtibial Transfemoral Desarticulação coxofemoral	46 (59,0) 31 (39,7) 1 (1,3)	6 (33,3) 11 (61,1) 1 (5,6)	40 (66,7) 20 (33,3) 0 (0,0)	0,010*

* p < 0,05; DP = desvio padrão.

 A média de idade dos participantes foi de 63,8 anos (DP 10,5), e 54 (69,2%) deles eram do sexo masculino. Pacientes submetidos a amputação maior que evoluíram para óbito apresentaram maior nível médio de creatinina sérica – 3,65 mg/dL [Desvio padrão (DP) 2,6] – e menor nível médio de hemoglobina sérica – 6,48 g/dL (DP 1,7) – quando comparados a pacientes submetidos a amputação maior que não morreram. Aproximadamente 53% dos pacientes foram internados apenas uma vez, enquanto 37 pacientes (47,4%) tiveram de ser internados mais de uma vez. A amputação transtibial foi realizada em 59,0% dos pacientes, e a amputação transfemoral foi realizada em 39,7%. Embora a maior parte dos pacientes (59,0%) tenha sido submetida a amputação transtibial, foi a amputação transfemoral (61,1%) que se associou ao óbito (p = 0,010). 

 Dos 78 pacientes, 68 (87,2%) tiveram culturas positivas, e 12,8% tiveram culturas negativas. Durante o período de internação considerado, 42 pacientes (53,9%) realizaram apenas uma cultura bacteriológica de tecido profundo, 21 (26,9%) realizaram duas culturas, e 15 (19,2%) realizaram mais de duas culturas. Entre os 68 pacientes com culturas positivas, 67,9% apresentaram o crescimento de apenas um microrganismo, enquanto 32,1% apresentaram o crescimento de dois ou mais microrganismos. A infecção hospitalar da úlcera foi registrada em 24 pacientes (30,8%). 

 Na Tabela 2 , estão descritos os gêneros de bactérias isolados nas culturas bacteriológicas de tecido profundo dessa amostra. As bactérias mais frequentes isoladas nas amostras dos pacientes que realizaram amputação maior e evoluíram para óbito foram *Acinetobacter* spp. (33,3%), *Morganella* spp. (33,3%) e *Proteus* spp. (27,8%). 

**Tabela 2:** Comparação entre os grupos de pacientes quanto aos gêneros de bactérias isoladas em culturas bacteriológicas de tecido profundo.

	**Total** **(n = 78)**	**Amputação maior e óbito** **(n = 18)**	**Amputação maior e não óbito** **(n = 60)**	**p**
Gêneros Gram-positivos				
*Enterococcus spp.* n (%)	15 (19,2)	3 (16,7)	12 (20,0)	0,999
*Staphylococcus spp.* n (%)	8 (10,3)	2 (11,1)	6 (10,0)	0,999
*Streptococcus spp.* n (%)	4 (5,1)	1 (5,6)	3 (5,0)	0,999
Gêneros Gram-negativos				
*Acinetobacter spp.* n (%)	19 (24,4)	6 (33,3)	13 (21,7)	0,312
*Citrobacter spp.* n (%)	4 (5,1)	1 (5,6)	3 (5,0)	0,999
*Escherichia spp.* n (%)	9 (11,5)	2 (11,1)	7 (11,7)	0,999
*Enterobacter spp.* n (%)	9 (11,5)	2 (11,1)	7 (11,7)	0,999
*Klebsiella spp.* n (%)	6 (7,7)	0 (0,0)	6 (10,0)	0,327
*Morganella spp.* n (%)	19 (24,4)	6 (33,3)	13 (21,7)	0,312
*Proteus spp.* n (%)	18 (23,1)	5 (27,8)	13 (21,7)	0,589
*Pseudomonas spp.* n (%)	13 (16,7)	2 (11,1)	11 (18,3)	0,721
*Serratia spp.* n (%)	4 (5,1)	1 (5,6)	3 (5,0)	0,999
*Stenotrophomonas spp.* n (%)	1 (1,3)	0 (0,0)	1 (1,7)	0,999

As frequências foram calculadas com base na amostra total de 78 pacientes.

 Na Tabela 3 , estão apresentados os resultados da análise univariada, demonstrando a significância para as variáveis: idade, níveis de creatinina ≥ 1,3 mg/dL, níveis de hemoglobina < 11 g/dL, amputação transfemoral. 

**Tabela 3:** Análise univariada das 23 variáveis clínicas, laboratoriais e bacteriológicas dos pacientes.

	***Odds ratio***	**Intervalo de confiança de 95%**	**p**
Idade, por ano	1,06	1,00-1,12	0,040 ^*^
Gênero, masculino	1,60	0,53-4,84	0,397
Creatinina ≥ 1,3 mg/Dl	15,9	1,99-127,2	0,009*
Hemoglobina < 11 g/dL	0,63	0,43-0,92	0,015*
Culturas positivas	3,00	0,35-25,44	0,314
Cultura polimicrobiana	1,08	0,35-3,31	0,894
Taxa de infecção hospitalar da úlcera	0,57	0,17-1,96	0,374
Reinternação	0,86	0,29-2,46	0,772
Amputação transfemoral	3,67	1,18-11,35	0,024*
*Acinetobacter spp.*	1,80	0,57-5,75	0,316
*Citrobacter spp.*	1,12	0,11-11,45	0,925
*Escherichia spp.*	0,95	0,18-5,02	0,948
*Enterococcus spp.*	0,80	0,20-3,22	0,753
*Enterobacter spp.*	0,95	0,18-5,02	0,948
*Klebsiella spp.* ^£^	---	---	---
*Morganella spp.*	1,81	0,57-5,75	0,316
*Proteus spp.*	1,39	0,42-4,62	0,590
*Pseudomonas spp.*	0,56	0,11-2,78	0,476
*Staphylococcus spp.*	1,13	0,21-6,13	0,892
*Serratia spp.*	1,12	0,11-11,45	0,925
*Stenotrophomonas spp.*	---	---	---
*Streptococcus spp.*	1,12	0,11-11,45	0,925

* p < 0,05;

*^£^*para *Klebisiella spp* e *Stenotrophomonas spp* não houve óbitos.

 Na Tabela 4 , estão apresentados os resultados obtidos com a análise multivariada, indicando os fatores associados ao óbito nesta amostra. 

**Tabela 4:** Análise multivariada das variáveis associadas ao óbito dos pacientes.

	***Odds ratio***	**Intervalo de confiança de 95%**	**p**
Creatinina ≥ 1,3 mg/dL	17,8	2,1-149,5	0,008
Amputação transfemoral	4,5	1,3-15,7	0,016

## DISCUSSÃO

 Neste estudo, foram investigados os fatores de risco associados a mortalidade em pacientes com pé diabético infectado submetidos a amputação maior. O nível de creatinina sérica igual ou maior a 1,3 mg/dL foi considerado fator de risco para óbito, assim como a amputação transfemoral. 

 Neste estudo, pacientes cujo nível de creatinina sérica foi igual ou superior a 1,3 mg/dL tiveram risco de óbito de quase 17,8 vezes em relação àqueles com nível de creatinina abaixo desse valor. Pacientes diabéticos com nefropatia desenvolvem úlcera nos pés na proporção duas vezes maior comparada com os demais diabéticos. Em pacientes com nefropatia diabética, a presença de úlcera nos pés é considerada uma das principais causas de morbidade. Esses pacientes possuem risco de amputação aumentado de 6,5 a 10 vezes, uma vez que o tempo de cicatrização da úlcera é prolongado com o aumento da creatinina sérica. Além disso, a mortalidade está associada a gravidade da nefropatia diabética 18 ^-^ 20 . 

 Dos 39,7% pacientes que realizaram amputação transfemoral, 61,1% evoluíram para óbito. Esses pacientes apresentaram risco de óbito de 4,5 vezes em relação àqueles que se submeteram a outros níveis de amputações. O paciente diabético tem risco 15 a 40 vezes maior de se submeter a amputação que o indivíduo não diabético. Pacientes que realizaram amputações transfemoral manifestam doenças sistêmicas graves, como insuficiência cardíaca, doença arterial coronariana, doença cerebrovascular, insuficiência renal e doença pulmonar obstrutiva crônica, entre outras. Dessa forma, cursam com alta mortalidade 21 . 

 O gênero bacteriano isolado em úlceras infectadas em pé diabético não foi fator de risco associado a mortalidade. Os gêneros bacterianos *Acinetobacter* spp. (33,3%), *Morganella* spp. (33,3%) e *Proteus* spp. (27,8%), pertencentes ao grupo de bactérias Gram-negativas, foram os mais isolados nas culturas dos pacientes submetidos a amputação maior que evoluíram para óbito. 

 Em um trabalho realizado no Hospital Central da Santa Casa de São Paulo, também se observou o isolamento de bactérias Gram-negativas, com 66% das culturas das úlceras profundas (Classificação de Wagner ≥ 3) dos pacientes isolando bactérias Gram-negativas 22 . 

 Na amostra estudada, 12,8% dos pacientes tiveram o resultado de cultura de tecido profundo negativo, ou seja, não houve isolamento de bactérias. Deve-se ressaltar que muitos dos pacientes estudados podem ter recebido tratamento prévio com antibacteriano em outras unidades de saúde, e o uso de antibacteriano antes da coleta do material biológico pode resultar em uma cultura negativa, mascarando o real resultado 8 . 

 Culturas polimicrobianas podem estar presentes em infecções de úlceras profundas em pacientes com pé diabético 11 . O paciente com infecção polimicrobiana apresenta processo de cicatrização da úlcera mais prolongado devido à presença de diversos fatores de virulência secretados pelas diferentes espécies de bactérias presentes na infecção 12 . Neste estudo, não houve predomínio de cultura polimicrobiana (32,1%), e essa variável não foi considerada como fator de risco para óbito. 

 Alguns fatores podem contribuir para a obtenção de culturas monomicrobianas. Pacientes com úlceras recentes, ou seja, com pouco tempo de evolução, apresentam isolamento monomicrobiano 23 . Como se trata de um estudo retrospectivo, não foi possível afirmar o tempo de evolução da úlcera dos pacientes, pois o dado não foi encontrado em seus prontuários eletrônicos. 

 O uso de antibacteriano pelos pacientes antes da coleta da amostra também pode levar ao isolamento de apenas bactérias resistentes ao antibacteriano, pois bactérias que forem sensíveis ao tratamento prévio serão eliminadas 23 . Muitos desses pacientes estudados foram admitidos na instituição já usando antibacteriano prescrito em unidades de saúde anteriores. 

 O resultado da cultura do material biológico pode ser influenciado caso seja realizada uma coleta inadequada na úlcera. A coleta mais adequada para análise de cultura seria de tecidos profundos livres de contaminação ou de necrose, visto que coletas de tecidos superficiais podem reduzir o número de patógenos isolados, resultando na obtenção de culturas monomicrobianas 24 . 

 Por fim, deve ser considerada a limitação do laboratório de microbiologia que não realiza culturas para anaeróbios estritos. As culturas para anaeróbios facultativos são solicitadas principalmente em casos de osteomielite. Nesta amostra, não foram encontrados registros de culturas de anaeróbios facultativos. Bactérias anaeróbias estão presentes em 15% das infecções graves de úlcera em pé diabéticos 11 . 

 Neste estudo, a taxa de infecção hospitalar da úlcera (30,8%) não foi fator de risco para óbito. Essas infecções podem ser associadas a contaminação por equipamentos hospitalares ou pelas mãos colonizadas da equipe profissional que tem contato direto com o paciente. 

 Nesta amostra, destacaram-se pacientes do sexo masculino (69,2%) e com média de idade superior a 60 anos. Pacientes que evoluíram a óbito (68,3 anos, DP 11,4) tiveram a média de idade maior quando comparados aos que sobreviveram (62,4 anos, DP 9,9) (p = 0,034). A idade avançada tem sido considerada um fator de risco para aumento de mortalidade hospitalar em pacientes com amputação maior precedida por úlcera nos membros inferiores 5 . 

 Os pacientes apresentaram taxa de reinternação de 47,4%, contudo, essa elevada taxa não foi associada a óbito. Na maior parte dos casos, o paciente retorna à instituição em consequência da complicação infecciosa relacionada ao coto da amputação 25 . 

 Este estudo teve algumas limitações. Primeiro, a falta de alguns dados era inevitável, considerando isso próprio dos estudos retrospectivos. O tempo de evolução da úlcera, considerado um dado importante, não foi coletado. Outro fato relevante foi a limitação do laboratório em realizar culturas de anaeróbios. Além disso, muitas coletas de tecidos foram realizadas em pacientes que poderiam estar em uso de antibacterianos previamente à internação. 

 Em conclusão, os níveis séricos de creatinina ≥ 1,3 mg/dL e amputação transfemoral se associaram a óbito em pacientes amputados por pé diabético com infecção.
